# Everybody's Page

**Published:** 1889-02-09

**Authors:** 


### Everybody's Page.

About 180,000 doses of castor-oil are annually consumed in Paris.

The Sanitary Board of Montreal has ordered that a placard bearing the word "Diphtheria" shall be affixed to the doors of all houses infected with that disease.

In an article entitled "Therapeutic Nihilism," which recently appeared in the *Boston Medical and Surgical Journal*, Dr. M. Clarke defines homœopathy as "that therapeutic opera bouffe,"

According to Dr. Bedoni, unglazed tissue paper, such as is used for the manufacture of cigarettes, is useful in the form of compresses composed of one or several sheets, and can also be used for bandages, or, in fine shavings, for lint.

The American *Journal of Pharmacy* recommends the following prescription for an antidote to poison, suitable for use when the poison is unknown: Equal parts of calcined magnesia, wood charcoal, and hydrated oxide of iron, mixed with a sufficient quantity of water.

The Brooklyn doctors are beginning to complain of the injury done to the members of the profession by the increasing number of dispensaries. At present there are twenty-three, and more are threatened. The complaint is, of course, that which we know so well on this side of the water, that patients who are able to pay manage to get treatment gratis.

Vaccination is compulsory in England and optional in France. In the large cities of France the number of deaths from small-pox was 1,956, or 0·31 per 1,000 of the living population. In the large cities of England during the same period the number of deaths was 332, or 0·04 per 1,000. Does not this say something for vaccination?

Empedocles, who committed suicide by throwing himself into the crater of Mount Etna, believed that the blood of man contained an infinite number of demons. Had Empedocles lived in our time he would probably have been a believer in the ubiquitous microbe. We may look upon him as the patriarch of bacteriologists.

An English doctor at Hong-Kong declares that the opium habit among the Chinese is not really more dangerous, when indulged in moderation, than that of tobacco-smoking, and that the evil is not to be compared with the abuse of alcohol. It may be due to the fact that concentrated forms of opium, such as laudanum and morphia, are in use here, but doctors in this country have not found the opium habit innocuous.

In Spain, contrary to our preconceived ideas, there are certain families, the *crême de la crême* of the nobility, who are marked by fair or red hair. They are the descendants of the conquering Visi-Goths, who descended on the Iberian peninsula in the fifth century, and are proud of the fair complexion that marks their descent from the strong old northern heroes.

Dr. Maraghano, of Genoa, recently addressed the Italian Medical Society on the treatment of pneumonia. He looks upon the rapid pulse which characterises that disease as one of its most serious symptoms, as indicating a strain on the heart which it is unable to bear. To sustain the flagging organ he recommends the free use of alcohol with digitalis, or, by preference, strophanthus when speedy action is required; and he also advises blood-letting as a mechanical relief to the pressure on the heart. It will be strange if, after all our horror of the barbarous doctoring of former days, we should return to bleeding as a cure for fevers. It will be the resurrection of Dr. Sangrado.

Albinos are deficient in colouring-matter throughout their bodies. Their flaxen hair shows this, and their eyes are pink because the iris is deficient in pigment to hide the red fundus from shining through. They suffer greatly from a strong light, because of this lack of colour to intercept it. They are relieved by wearing tinted glasses, which in some degree make up for the lack of natural pigment.

An idea has been started that fair-haired people are more liable to consumption than those of dark complexion. The explanation probably is that consumption is commonest in northern lands—a fact that the severer climate will account for—where the majority of the inhabitants are fair, but phthisis does not attack them in larger proportion to their numbers than their darker neighbours. And, in fact, the dark-skinned natives of the south are apt to be attacked by consumption when they come to our colder country.

Dr. Cassels, of Edinburgh, was a very strong advocate of nasal respiration, which he preached in a pamphlet bearing the sensational title, "Shut Your Mouth and Save Your Life." He quotes from Mr. George Catlin the dictum, "If I were to endeavour to bequeath to posterity the most important motto which human knowledge can convey, it should be in three words—'Shut your mouth.'" We suspect Mr. Catlin of a discreet *double entendre;* but it seems that Aristotle, who anticipated many of the discoveries of modern science, held that in breathing through the nose there is an action of the air on the sensorium at the base of the brain which promotes intelligence, and, in man, develops energetic and clear mental action.

Dr. Brown Sequard has been surprising the Société de Biologie by two statements which run counter to all we have been taught of the chemical ingredients of healthy air. He says that oxygen is not so necessary to respiration as is generally supposed, and that pure nitrogen, usually thought to be of little account, good only to weaken the oxygen, as water weakens wine, is an important element. He also thinks that the poisonous nature of carbonic acid gas has been greatly overstated. It is usually taught that air containing ten per cent. of carbonic acid is poisonous, but Dr. Brown Sequard, and a friend of his, M. D'Arsonal, felt no inconvenience from an atmosphere containing twenty per cent. It is possible that the really dangerous element in expired air is not carbonic acid, but albumenoid of ammonia.

A correspondent of *Tit-Bits*—by the way, surely that very popular newspaper might acknowledge the extracts it takes from our columns—writes to express the opinion that it would be better for the happiness of mankind if in marriages the wives were older than their husbands. Women, he says, live longer, on the average, than men; therefore, he says, "the chances of the lives of married couples closing simultaneously, or more nearly so, would be much increased if the wife were from five to ten years older than her husband." He also believes that the advice and guidance of a wife who has some independent experience of life would be of value to a man, and even goes so far as to say that "If only one is to rule, much better will it be for the husband and the household, where the woman rather than the man is supreme." In fact, he is almost as great a believer in the virtue of woman's rule as the gentleman who declared that he owed all his prosperity to his having, in defiance of the marriage service, obeyed his wife from his wedding day. It is rash to generalise on specifics for happy marriages. Many marriages are successful where the wife is the senior; on the other hand, many wives are miserably jealous of husbands younger than themselves.

